# An eleven autophagy-related genes-based prognostic signature for endometrial carcinoma

**DOI:** 10.1186/s43046-022-00135-2

**Published:** 2022-10-10

**Authors:** Shiyang Li, Junan Pan, Yanyu Zhang, Yan Tang, Xiaobing Zeng, Shihai Wang, Dengxuan Wu, Yuyong Liu, Dawen Xu, Jianjun Lan, Dong Hu

**Affiliations:** 1 Present Address: Division of Cardiology, Panzhihua Central Hospital, Panzhihua, China; 2 Clinical Lab, Panzhihua Central Hospital, Panzhihua, China; 3 The First Affiliated Hospital of the Medical College, Shihezi University, Shihezi, China; 4 Department of Cardiology, Renmin Hospital of Wuhan University, Wuhan, China

**Keywords:** Endometrial cancer, Prognosis, The Cancer genome atlas, Autophagy

## Abstract

**Background:**

Endometrial cancer (EC) is a common malignant tumor in women with increasing mortality. The prognosis of EC is highly heterogeneous which needs more effective biomarkers for clinical decision. Here, we reported the effect of autophagy-related genes (ARGs) on the prognosis of EC.

**Methods:**

The expression data of EC tissues and adjacent non-tumor samples were available from the TCGA dataset and 232 autophagy-related genes were from The Human Autophagy Database. A prognostic ARGs risk model was further constructed by using LASSO-Cox regression, and its prognostic and predictive value were evaluated by nomogram. Further functional analysis was conducted to reveal a significant signaling pathway.

**Results:**

A total of 45 differentially expressed ARGs were obtained, including 18 upregulated and 27 downregulated genes. Eleven ARGs (BID, CAPN2, CDKN2A, DLC1, GRID2, IFNG, MYC, NRG3, P4HB, PTK6, and TP73) were finally selected to build ARGs risk. This signature could well distinguish between the high- and low-risk patients (survival analysis: *P* = 1.18E-10; AUC: 0.733 at 1 year, 0.795 at 3 years, and 0.823 at 5 years). Furthermore, a nomogram was plotting to predict the possibility of overall survival and suggested good value for clinical utility.

**Conclusion:**

We established an eleven-ARG signature, which was probably effective in the prognostic prediction of patients with EC.

**Supplementary Information:**

The online version contains supplementary material available at 10.1186/s43046-022-00135-2.

## Introduction

According to current report [1], endometrial cancer (EC) has kept rising in both incidence and mortality, despite advances in treatment methods which is still sixth rank cancer in women globally [2]. As the aging population and increasing of obesity, the EC incidence is expected to rise even further [3]. The classic treatment strategy for EC involves surgery and adjuvant therapy based on final pathology for early-stage disease. Early detection of EC at early stages has a relatively favorable prognosis [4]. The prognosis of advanced EC remains poor because of its high rates of recurrent, metastatic [5]. In addition, the median survival time is less than 12 months for advanced EC [6].

The current known molecular markers of EC include PTEN, PI3K/AKT/mTOR pathway, Ras/Raf pathway, HRD pathway, and other genetic architecture such as ARID1A, CTNNB1, FGFR2, HER2/neu, and p53 which has been performed diagnostic, prognostic, and/or predictive of response to EC [7].

Autophagy plays a crucial role in cellular physiology and is responsible for degrading dysfunctional organelles, intracellular microbes, and pathogenic proteins by lysosome which could defeat deficiencies that may lead to disease [8]. Meanwhile, autophagy has been reported involved in nutrient recycling and metabolic adaptation and could regulate and act as double-edged sword in the development of cancer. Mountains of research have exposed that autophagy involves in the physiological and pathophysiological processes of endometrium stroma cells and epithelial cells and is associated with the EC. Triggering or inhibiting the molecular markers of autophagy including p53, AMPK, and PI3K/AKT/mTOR pathways could influence the process of EC development. Some reports found ATG7, ULK4, and other genes of somatic mutations different expressions in the EC [9]. Additionally, autophagy has been linked to resistance to chemotherapy [10]. Though some studies discussed the association between autophagy genes and EC [11], the target genes of autophagy need to be deeply practiced in clinical application. Up to now, only a few work analyzed the link between autophagy genes and progression of EC in small data [12], which need to be verified by other methods. In this study, we obtained the expression data of EC tissues and adjacent non-tumor samples from The Cancer Genome Atlas (TCGA) public database and constructed a prognostic model with eleven autophagy-related genes (ARGs), which could accurately assess the prognostic risk of patients with endometrial cancer.

## Method

### Data acquisition

A total of 370 uterine corpus endometrial carcinoma (EC) tissues and 11 adjacent non-tumor samples were obtained from UCSC Xena (https://xenabrowser.net/), including mRNA expression matrix, clinical, and survival information. We downloaded 232autophagy-related genes (ARGs) from The Human Autophagy Database (http://www.autophagy.lu/index.html).

### Clustering and differentially expressed ARGs analysis

The ARGs log-transformed values were used to perform tsne analysis using the R package “Rtsne” [13]. Then, “limma” package was used to identify the differentially expressed ARGs. Genes with fold change (FC) > 1 or < 0.5 and adjusted *P*-value < 0.05 were defined as differentially expressed genes [14].

### Functional pathway analysis

The webtool Metascape (http://metascape.org/gp/index.html#/main/step1) was used to perform functional enrichment of differentially expressed ARGs. Gene Ontology (GO) biological processes, Reactome gene sets, Canonical pathways, Wiki Pathways, and the Kyoto Gene and Genomic Encyclopedia (KEGG) were used to assess relevant functional categories. Enrichment pathways with *p* and *q* values less than 0.05 are considered as significant categories [15].

### Construction of prognostic signature based on ARGs

All DEGs were performed univariate Cox regression analysis to find out prognosis-related ARGs in EC. Then, significant genes (*p*-value < 0.05) were used to apply the least absolute shrinkage and selection operator (LASSO) Cox regression to narrow the range by using the “glmnet” R package [16]. The risk score formula was calculated as follows: risk score = (expressiongene1 × coefficientgene1) + (expressiongene2 × coefficientgene2) + … + (expressiongenen × coefficientgenen). Patients were classified into low-risk and high-risk groups based on the median value of risk scores. The Kaplan–Meier (K-M) method and time-dependent receiver operating characteristic (ROC) curve were generated to explore the prognostic accuracy of risk scores using the “survival” R package and “survivalROC” R package [17].

### Establishment and assessment of the nomogram

For better clinical application of the risk signature, other clinicopathologic parameters, including age, tumor stage, neoplasm histologic grade, and histological type, were analyzed to explore the diagnostic capability of multigene prognostic signature. According to the results of multiple Cox regression, we construct a nomogram that can evaluate the OS probability of 1, 3, and 5 years by the “rms” package. The calibration plot and concordance index (C-index) analysis were performed to validate the nomogram.

## Results

### Identification of differentially expressed ARGs

**Fig. 1:**
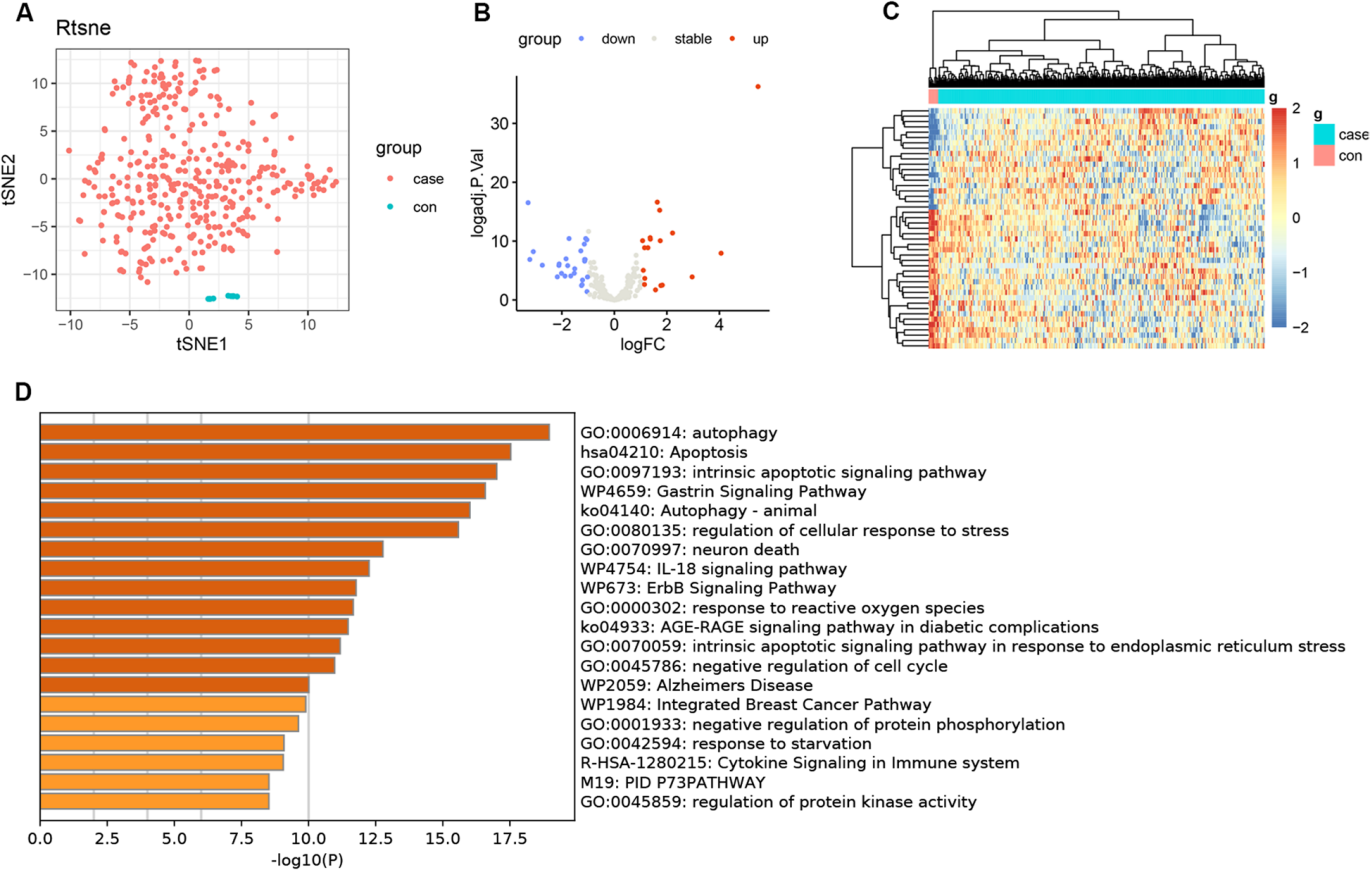
Identification of differentially expressed ARGs of EC. **A** Principal component analysis (PCA) between tumors and control samples; the result demonstrated the heterogeneity of the study. Differentially expressed ARGs in the study, 18 upregulated and 27 downregulated ARGs (|log2 FC|> 1, adjusted *P*-value < 0.05) was shown on **B** volcano plots and **C** hot map. Autophagy pathway, apoptosis pathway was major pathway by functional pathway analysis of ARGs was demonstrated on **D**

RNA-seq and clinical data from 370 EC tissue samples and 11 non-tumor samples were downloaded from TCGA, and ARGs were obtained from The Human Autophagy Database. As showed in Fig. 1A, ARGs appeared to have the potential ability to discriminate tumors from control samples. With criteria of |log2 FC|> 1 and adjusted *P*-value < 0.05, we finally obtained 45 differentially expressed ARGs, including 18 upregulated and 27 downregulated ARGs (Fig. 1B, C, Table S1). The pathway enrichment analysis indicated that above 45 genes participate in the autophagy pathway, apoptosis pathway, intrinsic apoptotic signaling pathway, and gastrin signaling pathway (Fig. 1D).

### Construction of prognostic prediction model

A total of 11 ARGs were identified significantly associated with survival status using univariate Cox regression analysis (*p* < 0.05, Table 1). We then calculated the relative regression coefficient of 11 ARGs using LASSO Cox regression model, which screened out eleven ARGs in final, including BID (BH3 interacting domain death agonist), CAPN2 (calpain 2), CDKN2A (cyclin-dependent kinase inhibitor 2A), DLC1 (DLC1 Rho GTPase-activating protein), GRID2 (glutamate ionotropic receptor delta type subunit 2), IFNG (interferon gamma), MYC (MYC proto-oncogene, bHLH transcription factor), NRG3 (neuregulin 3), P4HB (prolyl 4-hydroxylase subunit beta), PTK6 (protein tyrosine kinase 6), and TP73 (tumor protein p73). The final risk scores were as followers: risk score = (0.4585 * expression value of BID) + (− 0.0999 * expression value of CAPN2) + (0.0357 * expression value of CDKN2A) + (− 0.0445 * expression value of DLC1) + (0.1136 * expression value of GRID2) + (− 0.0968 * expression value of IFNG) + (0.0758 * expression value of MYC) + (0.1947 * expression value of NRG3) + (− 0.3470 * expression value of P4HB) + (0.1536 * expression value of PTK6) + (− 0.1034 * expression value of TP73).

**Table 1:** Univariate Cox regression analysis of the 11 genes

Gene	HR	95% CI	*P* value
BID	1.84	1.18–2.88	7.23E − 03
CAPN2	0.65	0.45–0.95	2.76E − 02
CDKN2A	1.22	1.09–1.37	7.87E − 04
DLC1	0.69	0.54–0.87	1.54E − 03
GRID2	1.22	1.07–1.4	3.41E − 03
IFNG	0.85	0.74–0.99	3.63E − 02
MYC	1.35	1.1–1.67	4.41E − 03
NRG3	1.38	1.23–1.55	1.77E − 08
P4HB	0.49	0.32–0.74	6.17E − 04
PTK6	1.15	1–1.31	4.43E − 02
TP73	0.76	0.67–0.87	4.67E − 05

**Fig. 2:**
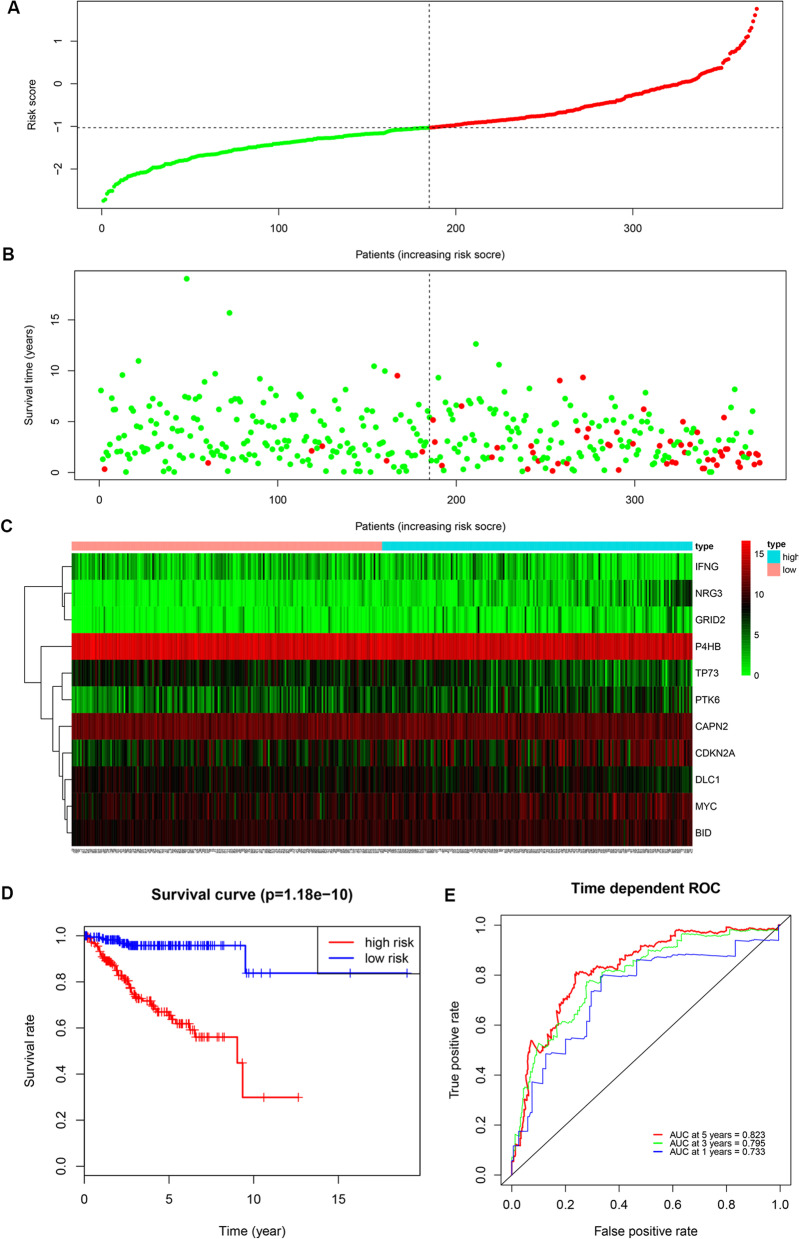
Construction of prognosis prediction model of EC. **A** Rank of risk score and distribution of groups. **B** The overall survival of patients in different groups. **C** Expression heatmap. **D** Kaplan–Meier survival analysis. **E** Time-dependent ROC curve analysis for survival prediction by the risk score

According to the median risk score in EC patients, 370 samples were divided into high-risk group (*N* = 185) and low-risk group (*N* = 185). K-M survival curves were used to analyze different survival times between high-risk and low-risk groups. The results showed that the high-risk group had a significantly poor prognosis (*p* = 1.18E − 10, Fig. 2A–D). Significantly, the area under the curve (AUC) of the corresponding receiver operating characteristic (ROC) curve for 1 year, 3 years, and 5 years of survival are 0.733, 0.795, and 0.823 respectively, which indicate that the risk score with11 ARGs has great ability in survival prediction of EC patients (Fig. 2E).

### Independence of the risk score from other clinical parameters

**Fig. 3:**
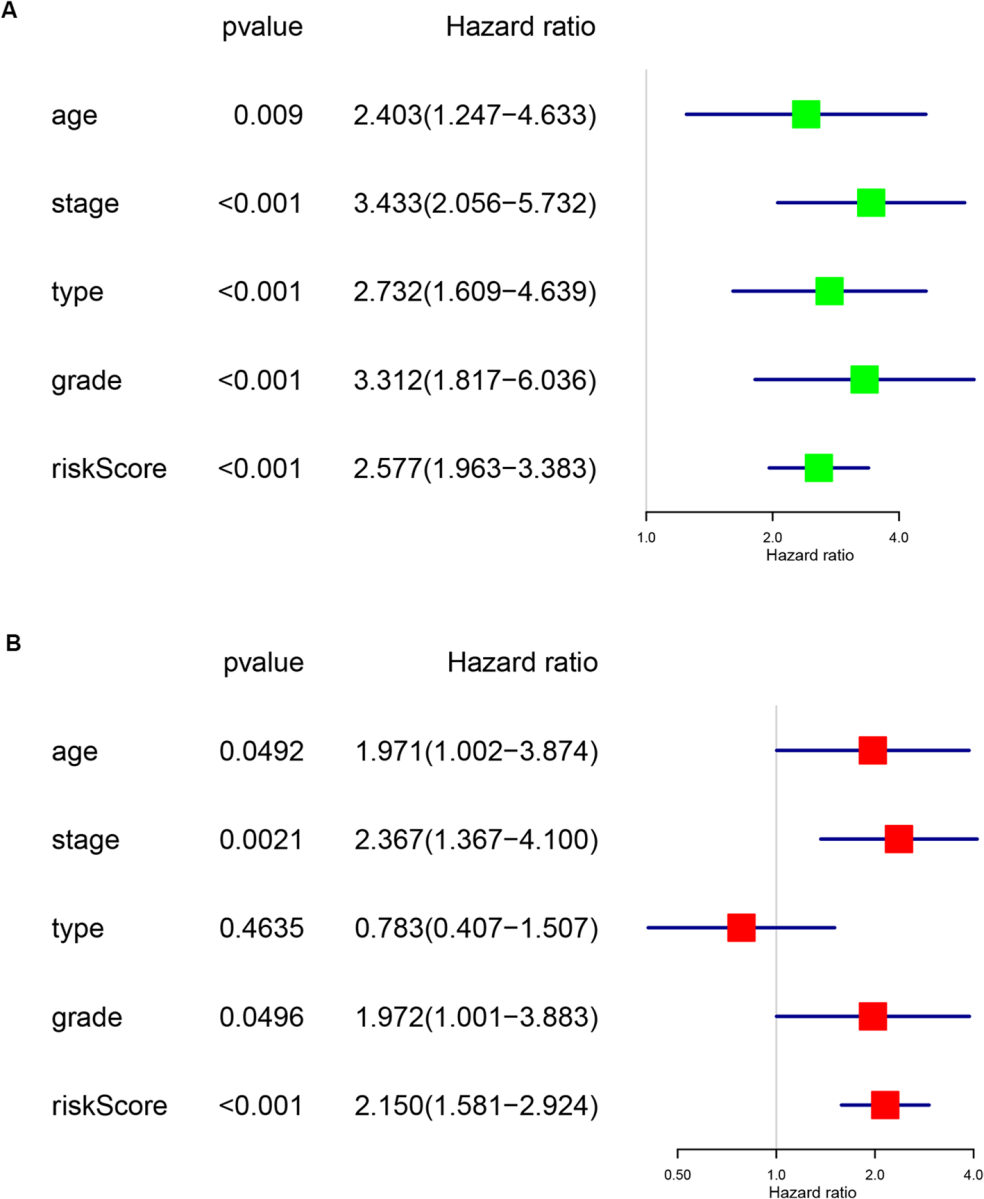
Univariate (**A**) and multivariate (**B**) regression analyses of the prognostic value for EC

In order to assess whether the risk score based on 11 ARGs is independent of other clinical variables including age, tumor stage, neoplasm histologic grade, and histological type, univariate and multivariate Cox regression analyses were performed. As showed in Fig. 3A, all clinical variables were significantly associated with the prognosis of EC (*p* < 0.05). Notably, the association between risk score and prognosis of EC patients remained after adjustment for four clinical values (*p* < 0.001, HR = 2.15, 95% CI = 1.58–2.92). Moreover, age (*P* = 0.049, HR = 1.97, 95% CI = 1.002–3.87), stage (*P* = 0.002, HR = 2.37, 95% CI = 1.37–4.1), and grade (*P* = 0.0496, HR = 1.97, 95% CI = 1.001–3.883) were also identified to be independent prognostic factors for EC (Fig. 3B).

### Construction of a nomogram for prediction 1-, 3-, and 5-year survival rate of EC

**Fig. 4:**
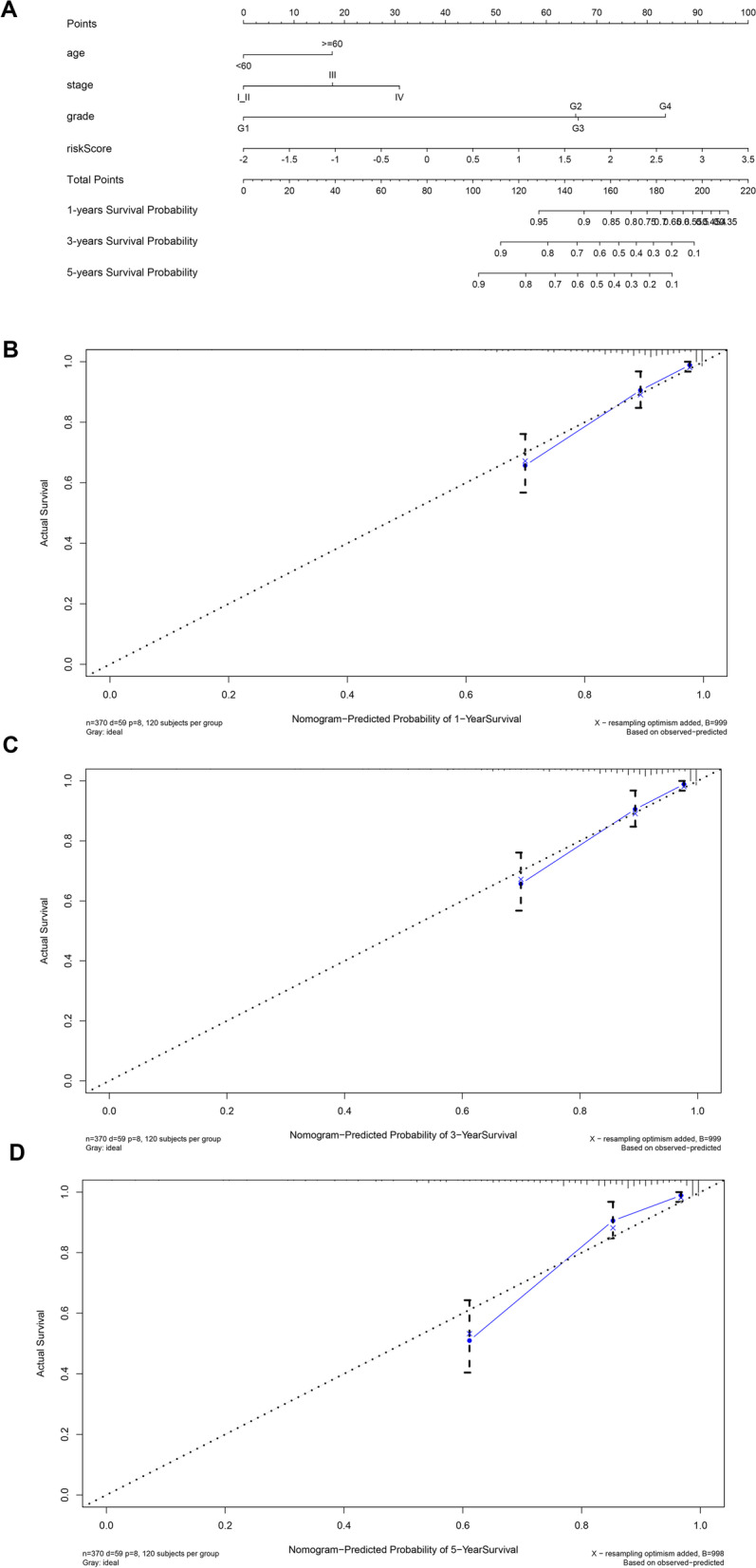
Construction of a nomogram for prediction survival of EC. **A** The nomogram to predict 1-, 3-, or 5-year survival rate. **B**–**D** The calibration plots for predicting patient 1-, 3-, or 5-year survival rate

For the convenience of clinical application, a clinically quantitative method was expected to build up to predict the 1-, 3-, and 5-year survival rate of EC. Based on our results, we combined ARGs risk scores with three clinical values (age, stage, and grade) to produce a nomogram (Fig. 4A), and the C-index reached up to 0.816. In the calibration curve, the diagonal line (ideal model) represented the best prediction (Fig. 4B–D), which suggested that the nomogram had the fine prediction ability.

## Discussion

Autophagy, engulf and resolve cellular components for delivery to the lysosome, is the highly conserved process. Autophagy-related (ATG) genes belong to evolutionarily conserved genes and represent potential targets for oncotherapy. Current effort of inhibiting the lysosome by using chloroquine will be used to inhibit tumor cell growth or induce tumor cell death [18]. The association between endometrial cancer survival and autophagy proteins had been reported in many studies. Lebovitz et al. [9] had explored the association of 211 human autophagy-associated genes with tumor and found core autophagy mutation such as ATG4 C, ULK4, and RB1CC1/FIP200 significantly correlated with EC. Besides, high expression of FAM83B was associated with a poor prognosis in endometrial cancer [19]. Increased BECN1 expression was found related to poor estimated 5-year survival [20]. Conversely, CDKN2A, an autophagy activator highly expressed in non-endometrioid tumors [21]. Together, the previous evidence suggested that the autophagy gene may involve in EC development.

In this study, we profiled the mRNA expression of 232 autophagy-associated genes in the TCGA endometrial carcinoma cohort. The risk score was calculated for each patient by integrating mRNA expression levels and risk coefficients for selected genes. Among them, a total of 45 differentially expressed ARGs were obtained, and 11 ARGs (BID, CAPN2, CDKN2A, DLC1, GRID2, IFNG, MYC, NRG3, P4HB, PTK6, and TP73) were finally selected to build ARGs risk by using LASSO Cox regression model. Zhang et al. developed a four-gene (CDKN2A, PTK6, ERBB2, and BIRC5) prognostic signature for EC [12], suggesting their potential as independent predictive biomarkers and therapeutic targets for endometrial cancer. Since then, autophagy gene signature has been frequently performed to predict the prognosis of EC. When we compared our gene signature with Zhang’s, two genes (CDKN2A, PTK6) were in common between the two datasets, suggesting that the key autophagy associated genes of EC may impact by different stages and subtypes of EC.

Besides, plenty of clinical and basic research had investigated the role of ARGs in endometrial carcinoma. CAPN2 [22], GRID2 [23], IFNG [24], MYC [25], NRG3 [23], P4HB [26], PTK6 [12], and TP73 [27] was reported linked to endometrial carcinoma and dynein light chain 1(DLC1) contributed to cell cycle progression in estrogen-stimulated cells and affect the progression of breast and endometrial cancer [28]. Recently, several lines of evidence reported that targeting autophagy is a therapeutic approach in endometrial cancer. Bortezomib, a 26S proteasome inhibitor used to treat multiple myeloma had anticancer properties by inhibiting the NF-κB pathway [29]. Paclitaxel exposure in endometrial cancer cell lines will decrease p62 abundance [30].

Lastly, we developed a nomogram to predict individuals’ clinical outcomes. Nomogram is a useful tool to measure risk on an individual basis by combining and delineating risk factors, which has been used in endometrial carcinoma patients [31, 32]. A nomogram statistics risk factor (age, gender, stage) for the predictive model was offered in presenting graph. For expected to conduct that could predict the 1-, 3-, and 5-year survival rate of EC. We merge ARGs risk scores and three clinical values (age, stage, and grade) to produce a nomogram, and the C-index were 0.816 accurately. The combination group of the autophagy-gene signature and prognostic factors achieved better prognostic performance. Genomic and bioinformatics technologies have provided pioneering insight into the molecular etiology of endometrial tumors [33]. Moreover, according to molecular prognostic value of PORTEC-3 trial, sort the high-risk of EC. The recurrence-free survival with adjuvant chemotherapy and radiotherapy for p53 abnormal tumors was significantly higher compared to other subgroups, regardless of histologic type [34]. Despite the attempt for integrating gene markers and traditional risk factors in predicting the prognosis of EC, the usage in the clinical trial is striking limited. Briefly, we indicated that a nomogram, including an 11-autophagy gene signature, could well predict 1-, 3-, and 5-year survival possibilities of EC patients in the current study.

This study has several limitations. First, our results are based on a small sample size and retrospective analysis, which need confirmation in further cohorts. In addition, these findings were deduced from TCGA, which required clinical trials to practice. Furthermore, it needs additional biological experiments to demonstrate results.

## Conclusion

We constructed a risk score with 11-autophagy related genes based on endometrial carcinoma of TCGA. And this risk score could independently predict the prognosis of EC patients. A nomogram combining gene signature and clinical risk factors could accurately predict the 1-, 3-, and 5-year survival probability for endometrial carcinoma patients. Our finding suggests that the 11-autophagy gene signature may help facilitate personalized medicine in the clinical setting.

## Supplementary Information

**Additional file 1:** 

## Data Availability

Data are available on reasonable request. All data relevant to the study are included in the article or uploaded as supplementary information. The authors declare that the research was conducted in the absence of any commercial or financial relationships that could be construed as a potential conflict of interest.

## Abbreviations

EC: Endometrial carcinoma
ARGs: Autophagy-related genes
KEGG: Kyoto Gene and Genomic Encyclopedia
LASSO: Least absolute shrinkage and selection operator
K-M: Kaplan-Meier
ROC: Receiver operating characteristic
C-index: Concordance index
BID: BH3 interacting domain death agonist
CAPN2: Calpain 2
CDKN2A: Cyclin-dependent kinase inhibitor 2A
DLC1: DLC1 Rho GTPase-activating protein
GRID2: Glutamate ionotropic receptor delta type subunit 2
IFNG: Interferon gamma
MYC: MYC proto-oncogene
bHLH: Transcription factor
NRG3: Neuregulin 3
P4HB: Prolyl 4-hydroxylase subunit beta
PTK6: Protein tyrosine kinase 6
TP73: Tumor protein p73
